# LOX-1 Activation by oxLDL Induces AR and AR-V7 Expression via NF-κB and STAT3 Signaling Pathways Reducing Enzalutamide Cytotoxic Effects

**DOI:** 10.3390/ijms24065082

**Published:** 2023-03-07

**Authors:** Felix Duprat, Catalina Robles, María Paz Castillo, Yerko Rivas, Marcela Mondaca, Nery Jara, Francisco Roa, Romina Bertinat, Jorge Toledo, Cristian Paz, Iván González-Chavarría

**Affiliations:** 1 Laboratory of Lipoproteins and Cancer, Pathophysiology Department, Universidad de Concepción, Concepción 4030000, Chile; 2 Pharmacology Department, Universidad de Concepción, Concepción 4030000, Chile; 3 Centro de Microscopía Avanzada, CMA-BIO BIO, Facultad de Ciencias Biológicas, Universidad de Concepción, Concepción 4030000, Chile; 4 Biotechnology and Biopharmaceuticals Laboratory, Pathophysiology Department, Universidad de Concepción, Concepción 4030000, Chile; 5 Laboratory of Natural Products & Drug Discovery, Center CEBIM, Department of Basic Sciences, Universidad de La Frontera, Temuco 4780000, Chile

**Keywords:** prostate cancer, castration-resistant prostate cancer, oxLDL, LOX-1, enzalutamide

## Abstract

The oxidized low-density lipoprotein receptor 1 (LOX-1) is one of the most important receptors for modified LDLs, such as oxidated (oxLDL) and acetylated (acLDL) low-density lipoprotein. LOX-1 and oxLDL are fundamental in atherosclerosis, where oxLDL/LOX1 promotes ROS generation and NF-κB activation inducing the expression of IL-6, a STAT3 activator. Furthermore, LOX-1/oxLDL function has been associated with other diseases, such as obesity, hypertension, and cancer. In prostate cancer (CaP), LOX-1 overexpression is associated with advanced stages, and its activation by oxLDL induces an epithelial-mesenchymal transition, increasing angiogenesis and proliferation. Interestingly, enzalutamide-resistant CaP cells increase the uptake of acLDL. Enzalutamide is an androgen receptor (AR) antagonist for castration-resistant prostate cancer (CRPC) treatment, and a high percentage of patients develop a resistance to this drug. The decreased cytotoxicity is promoted in part by STAT3 and NF-κB activation that induces the secretion of the pro-inflammatory program and the expression of AR and its splicing variant AR-V7. Here, we demonstrate for the first time that oxLDL/LOX-1 increases ROS levels and activates NF-κB, inducing IL-6 secretion and the activation of STAT3 in CRPC cells. Furthermore, oxLDL/LOX1 increases AR and AR-V7 expression and decreases enzalutamide cytotoxicity in CRPC. Thus, our investigation suggests that new factors associated with cardiovascular pathologies, such as LOX-1/oxLDL, may also promote important signaling axes for the progression of CRPC and its resistance to drugs used for its treatment.

## 1. Introduction

Lipoproteins are macromolecular complexes that transport lipids in a soluble form through blood circulation [1]. Lipoproteins are classified into five classes: chylomicrons (Quis), very low-density lipoproteins (VLDLs), intermediate-density lipoproteins (IDLs), low-density lipoproteins (LDLs), and high-density lipoproteins (HDLs) [2]. Human LDL is the primary lipoprotein for delivering exogenous cholesterol into the cells. High serum levels of LDLs are closely related to the development and progression of cardiovascular pathologies, such as endothelial dysfunction and atherosclerosis [3]. For years it was postulated that LDLs themselves promoted the development and progression of atherosclerotic plaques [4]. However, a simple observation changed this paradigm by showing that foam cells in the fatty streak of atherosclerotic plaque endocytose, were an oxidized form of LDLs (oxLDLs), not LDLs [5]. Then, other studies demonstrated that atherosclerotic plaques present high levels of oxLDLs, which undergo endocytosis via scavenger receptors that induce endothelial dysfunction and the formation of foam cells, two critical events in the progression of atherosclerosis [6]. The scavenger receptor LOX-1 is one of the most important receptors for oxLDLs, and its activation induces NADPH oxidase activity and reactive oxygen species (ROS) production, which can activate p38MAPK, ERK1/2, PI3K, NF-κB, and STAT3 signaling pathways that drive the expression of proinflammatory cytokines and the atherosclerosis progression [7,8,9,10]. Thus, it has been described that LOX-1 and oxLDLs are essential modulators of endothelial dysfunction, which is characterized by the increase in the secretion of pro-inflammatory cytokines, an increase in the ROS levels, the expression of pro-coagulants molecules, expression of adhesion molecules, such as I-CAM, V-CAM, E-selectin, and P-selectin, and an increase in endothelium permeability [11,12,13]. Furthermore, LOX-1 and oxLDLs promote macrophage activation and actively participate in foam cell formation. These cells are the main component of fatty streaks and are generated by oxLDL endocytosis through scavenger receptors, such as LOX-1, CD36, SR-B1, and SR-A1 expressed in the membrane of macrophages that transmigrate to the tunica intima of the arteries in response to the proinflammatory process. Finally, oxLDL/LOX-1 and other scavenger receptors participate in the generation of complicated atherosclerotic plaque through matrix metalloproteinases secretion (MMP2 and MMP9) mediated by activated macrophages present in atherosclerotic plaque [14,15,16,17]. However, the role of LOX-1 and oxLDLs is not only restricted to pathologies, such as atherosclerosis or endothelial dysfunction, but has also been associated with the progression of chronic pathologies, such as obesity [18,19], type II diabetes mellitus [20,21], and various types of cancer. The role of LOX-1 in cancer is related to the hallmarks of cancer, such as angiogenesis, invasion, metastasis, resisting cell death, and sustained proliferative signaling between others [22,23,24,25,26,27,28,29,30,31,32], and has been addressed in depth in several reviews [20,33,34,35,36].

Prostate cancer (CaP) is the most prevalent malignancy among adult men in the developed world [37]. Advanced CaP is treated with androgen deprivation therapy (ADT), which reduces the systemic androgen concentration, which is critical for the proliferation and survival of tumor CaP cells [38]. However, the patients in ADT will eventually develop castration-resistant prostate cancer (CRPC), which does not respond to ADT [39,40,41]. The androgen receptor (AR) antagonist enzalutamide is used for CRPC treatment, but a significant percentage of CRPC patients develop resistance to this drug in a short period of time [42]. The decrease in enzalutamide cytotoxic effects is associated with the activation of NF-κB and STAT3 signaling pathways, which promote the overexpression and activation of AR and AR-V7 and the secretion of cytokines, such as IL-6, that strengthen the activation of STAT3 signaling [43,44,45]. Thus, the overexpression of AR and AR-v7 decreased the cytotoxic and anti-proliferative effects of enzalutamide, inducing resistance against this drug.

High concentrations of oxLDLs were observed in patients with advanced CaP, and the overexpression of LOX-1 has been associated with high Gleason scores and clinicopathological stages III and IV [31,32]. Notably, oxLDL/LOX-1 induce angiogenesis, proliferation, and EMT in CaP cell lines and increase their tumorigenic potential. Furthermore, LOX-1 expression is necessary for tumor growth of CaP cell xenografts [32]. Interestingly, enzalutamide resistant LNCaP cells increase the uptake of acetylated LDLs (acLDLs) [46]. The acLDLs and oxLDLs are not recognized by LDL receptors, but instead by scavenger receptors, such as LOX-1 [47]. Thus, although some functions of LOX-1 and oxLDLs have been reported in CaP, the role of LOX-1 and oxLDLs in the expression of AR and AR-v7, the activation of downstream signaling pathways, and their impact on AR-targeting drugs have not been studied. In the present study, we demonstrate that the activation of LOX-1 by oxLDLs increases ROS production and activates NF-κB. The activation of NF-κB induces IL-6 secretion and STAT3 activation, which in turn promotes the overexpression of AR and AR-V7, and, therefore, decreases enzalutamide cytotoxicity in CRPC cells lines.

## 2. Results

### 2.1. LOX-1 Activation by oxLDLs Increases ROS Generation in CRPC Cells

**Figure 1:**
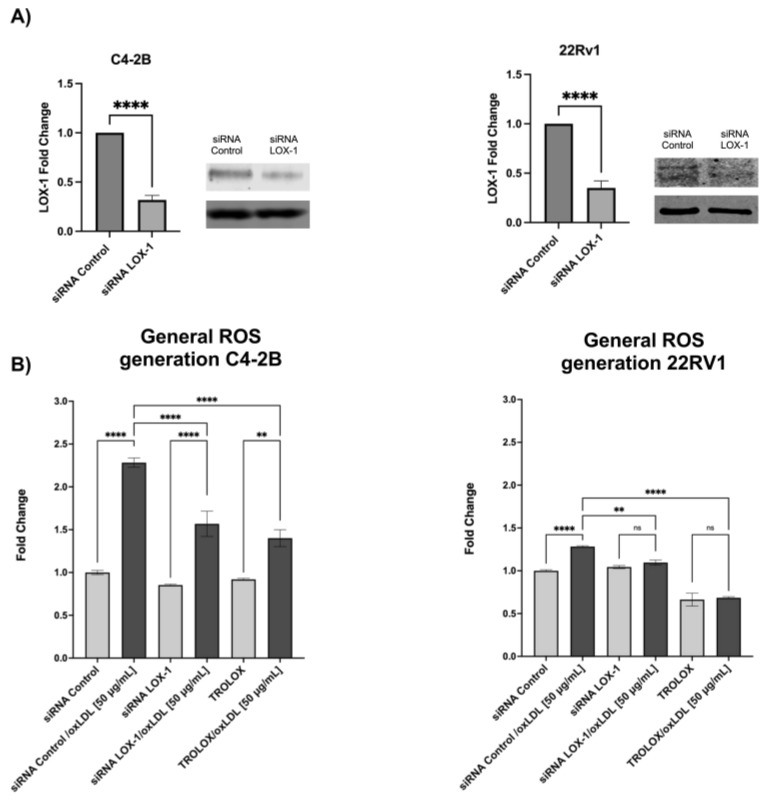
(**A**) Western blot of LOX-1 expression in CRPC cells, C4-2B and 22RV1 transfected with siRNA against LOX-1. To silence the LOX-1 RNA expression, C4-2B and 22RV1 prostate cancer cell lines were transfected with siRNA-LOX-1 or siRNA-control for 48 h, and the expression of LOX-1 was analyzed by Western blot. (**B**) OxLDL/LOX-1 induces ROS generation in castration-resistant prostate cancer cell models. C4-2B and 22Rv1 were transfected with a siRNA against LOX-1 or the control for 48 h or treated with 25 μM Trolox and then treated with 50 μg/mL oxLDL, and the ROS generation was analyzed 3 h post-treatment. The data represent the means ± S.D. of three independent experiments analyzed by one-way analysis of variance and Dunnett’s post-test or *t*-test for LOX-1 expression analysis (**** *p* ≤ 0.0001, ** *p* ≤ 0.01, ns: non-significant statistical difference).

LOX-1 activation by oxLDLs increases ROS generation in different cellular models associated with atherosclerosis progression, and has been described as an important signal for NF-κB activation [48]. To evaluate whether LOX-1 activation by oxLDLs promotes ROS generation in CRPC, C4-2B and 22RV-1 cells were transfected with siRNA LOX-1 or siRNA control (Figure 1A) and treated with or without 25 μM Trolox for 48 h, and then the cells were incubated with 50 μg/mL oxLDLs for 3 h. The results showed that oxLDLs significantly increased ROS generation by 2.3- and 1.3-fold in siRNA control C4-2B and 22Rv1 cells, respectively. However, in LOX-1 knockdown C4-2B and 22Rv1 cells, the effect of oxLDLs was prevented to similar levels to those observed in Trolox-treated cells (Figure 1B) suggesting that LOX-1 activation by oxLDLs induces ROS regeneration in C4-2B and 22Rv1 cells. 

### 2.2. OxLDL/LOX-1 Induces the Activation of NF-κB in C4-2B and 22RV1 Cells

**Figure 2:**
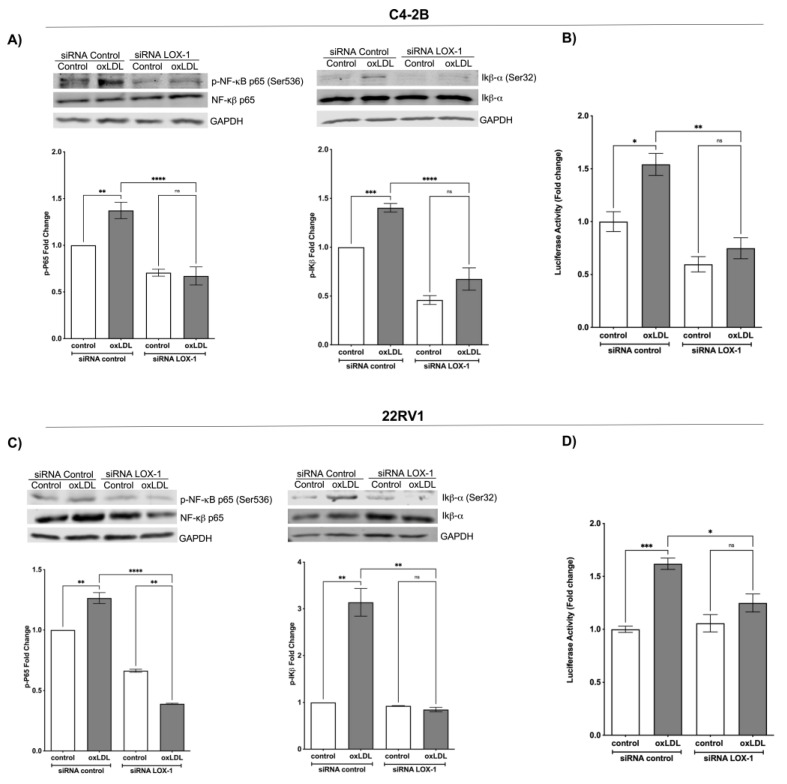
OxLDL/LOX-1 induces the activation of NF-κB in CRPC cell models. Western blot analysis of p-p65 and p-IκB-α in protein extracts of (**A**) C4-2B and (**C**) 22Rv1 cells transfected with siRNA against LOX-1 or control and treated with 50 μg/mL oxLDLs for 1 h. The NF-κB activity was analyzed using the reporter plasmid pHAGE NFKB-TA-LUC-UBC-dTomato-W in (**B**) C4-2B or (**D**) 22RV1 cells, co-transfected with siRNA control or siRNA LOX-1 and treated with 50 μg/mL oxLDLs for 24 h. The data represent the means ± S.D. of three independent experiments analyzed by one-way analysis of variance and Dunnett’s post-test (**** *p* ≤ 0.0001, *** *p* ≤ 0.001, ** *p* ≤ 0.01, * *p* ≤ 0.05, ns: non-significant statistical difference).

To determine the effect of LOX-1 activation by oxLDLs on the NF-κB signaling pathway, C4-2B and 22RV1 cells transfected with siRNA control or siRNA LOX-1 were treated with 50 μg/mL oxLDLs for 1 h, and the phosphorylation of p65 and IκB-α were analyzed by Western blot. The results showed that oxLDL induces p65 phosphorylation by 1.37- and 1.33-fold on C4-2B and 22RV1 cells (siRNA control), respectively. In IκB-α, the oxLDL induces the phosphorylation by 1.40- and 3.19-fold on C4-2B and 22RV1 (siRNA control) cells, respectively. In contrast, the oxLDL-induced phosphorylation of p65 and IκB-α was prevented in the LOX-1 knockdown C4-2B and 22RV1 cells. This suggests that activation of LOX-1 by oxLDLs induces the NF-κB signaling pathway activation (Figure 2A–C). To confirm these results, we evaluated the NF-κB activity using the reporter plasmid pHAGE NFKB-TA-LUC-UBC-dTomato-W on C4-2B or 22RV1 cells co-transfected with siRNA control or siRNA LOX-1 and treated with oxLDLs. The results showed that 50 μg/mL of oxLDLs increased the p65-luciferase reporter activity in siRNA control cells by 1.54- and 1.70-fold compared with C4-2B and 22RV1 (siRNA control) untreated cells. The LOX-1 knockdown C4-2B and 22RV1 cells exhibited low basal p65 promoter activity, and the effects of oxLDLs on p65 activity were prevented (Figure 2B–D). 

### 2.3. Activation of LOX-1 by oxLDLs Induces the Secretion of IL-6 and STAT3 Activation in C4-2B and 22Rv1 Cells

**Figure 3:**
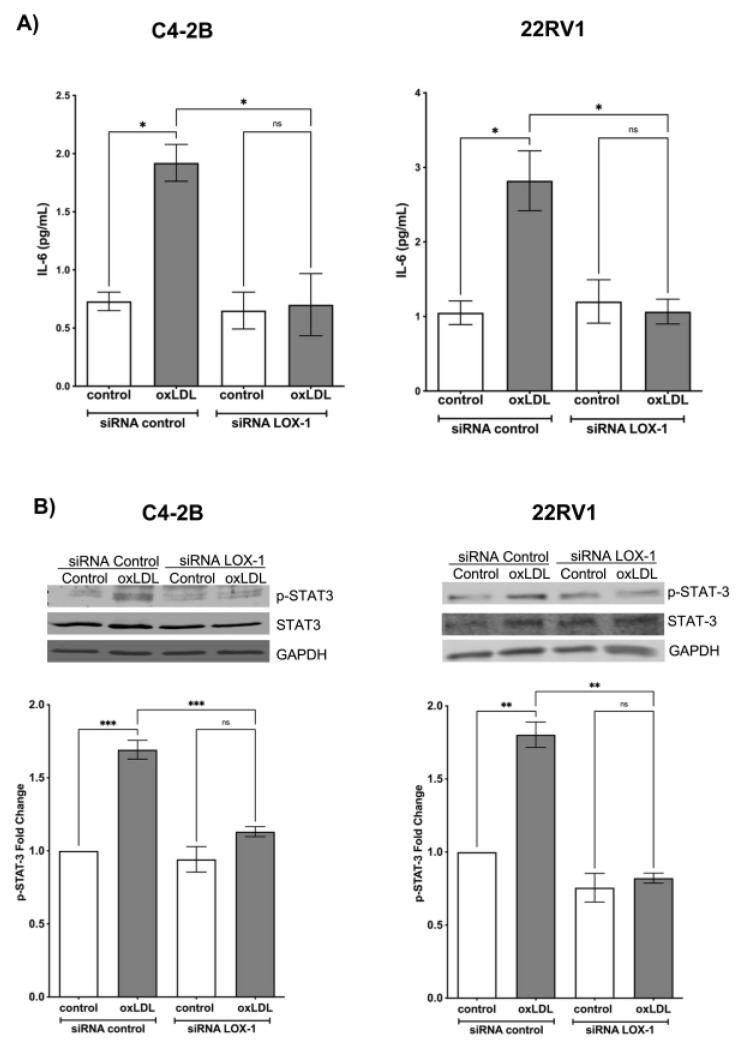
OxLDL/LOX-1 induces IL-6 secretion and STAT3 activation in CRPC cell models. (**A**) IL-6 ELISA assay from culture supernatants of C4-2B and 22Rv1 cells transfected with siRNA against LOX-1 or control and treated with 50 μg/mL oxLDLs for 24 h. (**B**) OxLDLs/LOX-1 induce the activation of STAT3 in CRPC cell models. Western blot analysis of p-STAT3 in protein extracts of C4-2B and 22Rv1 cells transfected with siRNA against LOX-1 or siRNA control treated with 50 μg/mL oxLDLs for 24 h. The data represent the means ± S.D. of three independent experiments analyzed by one-way analysis of variance and Dunnett’s post-test (*** *p* ≤ 0.001, ** *p* ≤ 0.01, * *p* ≤ 0.05, ns: non-significant statistical difference).

**Figure 4:**
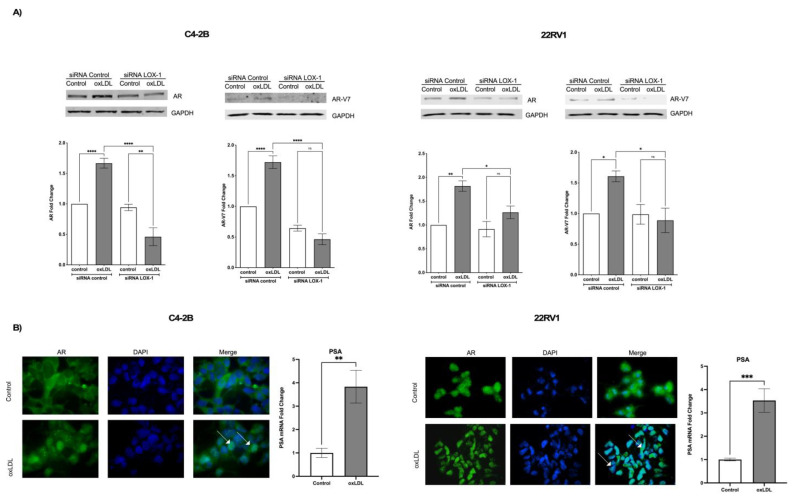
OxLDL/LOX-1 induces the expression of AR and AR-V7 on C4-2B and 22RV1 cells. (**A**) Western blot analysis of AR and AR-V7 in protein extracts of C4-2B and 22RV-1 cells transfected with siRNA against LOX-1 and treated with 50 μg/mL oxLDLs for 24 h. (**B**) OxLDL promotes AR nuclear translocation and PSA mRNA expression in C4-2B and 22RV1 cells. The white arrows indicate nuclear immunodetection of AR in oxLDL-treated C4-2B or 22RV1 cells. The data represent the means ± S.D. of three independent experiments analyzed by one-way analysis of variance and Dunnett’s post-test o *t*-test for PSA expression analysis (**** *p* ≤ 0.0001, *** *p* ≤ 0.001, ** *p* ≤ 0.01, * *p* ≤ 0.05, ns: non-significant statistical difference).

NF-κB activation significantly increases the expression and secretion of pro-inflammatory cytokines, such as IL-6, which has been described as an important cytokine in CRPC generation and enzalutamide resistance [49,50]. To determine the effect of LOX-1 activation on IL-6 secretion, C4-2B and 22RV1 cells transfected with siRNA control or siRNA LOX-1 were treated with 50 μg/mL oxLDLs for 24 h, and IL-6 levels were measured in the supernatants by ELISA. The results showed that oxLDL induces an increase in IL-6 concentration from 0.8 to 1.9 pg/m and 1.1 to 2.8 ng/mL in siRNA control C4-2B and 22Rv1 (Figure 3A). However, the effects of oxLDLs on the IL-6 secretion were prevented in the LOX-1 knockdown in C4-2B and 22RV1 cells (Figure 3A). Therefore, LOX1 activation by oxLDLs promotes the secretion of IL-6, probably through NF-κB activation in CRPC cell lines. IL-6-induced STAT3 activation partially promotes the resistance to enzalutamide in CRPC through AR and AR-V7 overexpression [51]. Given that LOX-1 activation by oxLDLs stimulated the secretion of IL-6 in our CRPC cell models, we analyzed the effect of oxLDLs on the STAT3 signaling pathway. C4-2B and 22RV1 cells were transfected with control siRNA or LOX-1 siRNA for 48 h, and then were treated with 50 μg/mL oxLDLs for 24 h, and the phosphorylation of STAT3 was determined by Western blot. The results showed that oxLDL increases STAT3 phosphorylation by 1.6- and 1.7-fold in siRNA control C4-2B and 22RV1 cells compared to untreated cells (Figure 4A). In LOX-1 knockdown in C4-2B and 22RV1 cells and the phosphorylation of STAT3 was prevented, suggesting that activation of LOX-1 by oxLDLs activates STAT3 signaling (Figure 3B).

### 2.4. LOX-1/oxLDL Induces AR and AR-V7 Overexpression in C4-2B and 22RV1 Cells

Overexpression and increased activity of AR and its splicing variant AR-V7 have been widely associated with CRPC and enzalutamide resistance [52]. In the case of AR-V7, is one of the most relevant variants in CRPC, responsible for the constitutive activation of AR signaling, even in the absence of a ligand [53]. In contrast to the native AR, AR-V7 lacks the ligand (DHT) binding site at which enzalutamide acts in an antagonistic fashion [54]. The clinical relevance of AR-V7 arises from a comparison between AR-V7 negative and positive patients in which the latter showed a lower survival rate. Indeed, 39% of patients with metastatic CRPC treated with enzalutamide express AR-V7 in the tumoral cells [52,55]. In turn, the presence of AR-V7 is associated with 9–15% of patients with a higher probability of generating enzalutamide resistance [52,55,56].

**Figure 5:**
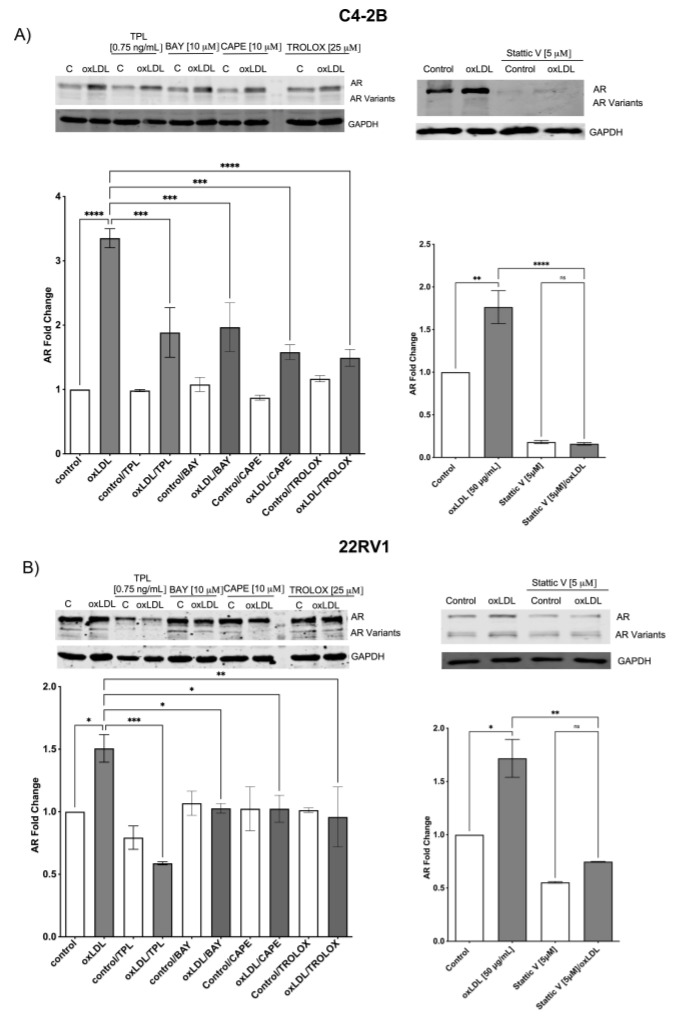
Inhibition of ROS generation, NF-κB, and STAT3 prevent oxLDL-induced AR expression. (**A**) C4-2B and (**B**) 22Rv1 cells were incubated with or without Trolox, triptolide (TLP), bay 11-7082 (BAY), caffeic acid phenethyl ester (CAPE), or Stattic (STAT3 inhibitor), then were treated with 50 μg/mL oxLDLs for 24 h and AR expression was evaluated by Western blot. The data represent the means ± S.D. of three independent experiments analyzed by one-way analysis of variance and Dunnett’s post-test (**** *p* ≤ 0.0001, *** *p* ≤ 0.001, ** *p* ≤ 0.01, * *p* ≤ 0.05, ns: non-significant statistical difference).

The mechanism associated with AR and AV-V7 overexpression has been correlated with NF-κB and STAT-3 activation, to this part, our data suggest that oxLDLs through LOX-1 induce ROS/NF-KB/IL-6 and STAT3 axis, which indicates that AR and AR-v7 could be overexpressed by LOX-1/oxLDL. To determine this, 22RV1 cells were transfected with siRNA control and siRNA LOX-1 for 48 h and then treated with 50 μg/mL of oxLDLs. The expression of AR and AR-V7 was analyzed by Western blot, the AR activity was analyzed through its translocation to the nucleus by immunocytochemistry assay, and the expression of prostate-specific antigen (PSA, gen under AR control) was analyzed by real-time PCR. The results showed that oxLDL induces by 1.65- and 1.7-fold the AR and AR-V7 expression in C4-2B (siRNA control) cells, respectively, and by 1.75- and 1.60-fold in 22RV1 (siRNA control) cells, respectively, compared with untreated cells. The effect of oxLDL-induced overexpression of AR and AR-V7 expression was prevented in LOX-1 knockdown C4-2B and 22RV1 cells (Figure 4A). Furthermore, oxLDL treatment in non-transfected cells induced the translocation of AR to the nucleus and increased PSA mRNA levels by 2.5- and 3.6-fold in C4-2B and 22RV1 cells, respectively (Figure 4B). Overall, these data suggest that oxLDLs not only induce the expression of AR and AR-V7 but also activate them in these CRPC cell lines, probably by ROS/NF-KB/IL-6 and STAT3 axis. To analyze whether the AR expression induced by oxLDLs is mediated by ROS, NF-κB, and STAT3 activation, we used a ROS inhibitor (Trolox), three NF-κB inhibitors: triptolide (TLP), bay 11-7082 (BAY), caffeic acid phenethyl ester (CAPE), and a chemical inhibitor of STAT3 activity Stattic. C4-2B and 22RV1 cells were co-treated with 25 μM Trolox, 0.750 ng/mL TPL, 10 μM BAY, 10 μM CAPE, or 5 μM Stattic and 50 μg/mL oxLDLs for 24 h, and the expression of AR was analyzed by Western blot. The results showed that Trolox, BAY, CAPE, and Stattic prevent oxLDL-induced AR overexpression in both CRPC cell lines (Figure 5A,B). Moreover, the results show that in C4-2B cells, STAT-3 inhibition generates a significant decrease in AR expression (Figure 5A). Similarly, inhibition of NF-κB by TPL in 22RV1 cells decreases the AR expression levels (Figure 5B).

### 2.5. LOX-1 Activation by oxLDLs Decreases the Cytotoxic Effects of Enzalutamide on C4-2B and 22RV1 Cells

**Figure 6:**
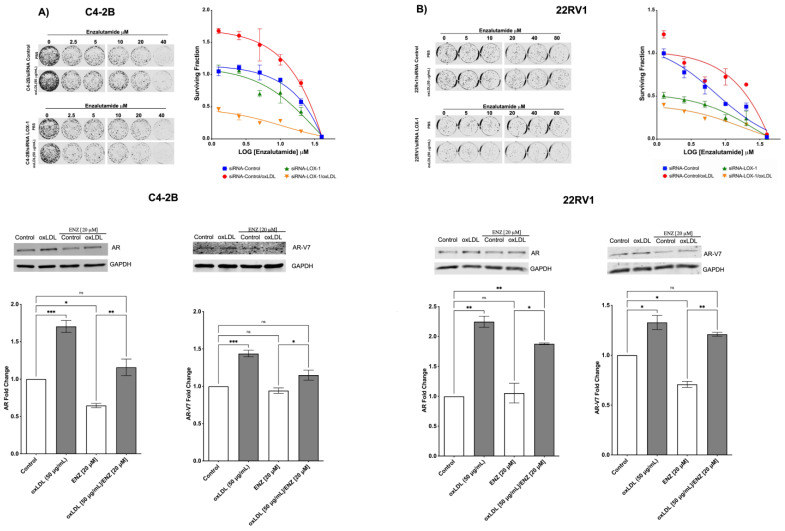
OxLDL/LOX-1 prevents the enzalutamide effects in CRPC cell lines. (**A**) clonogenic assay of C4-2B and 22Rv1 cells transfected with a siRNA against LOX-1 or siRNA control and co-treated with enzalutamide [0–40 μM] and oxLDL 50 μg/mL oxLDL. (**B**) oxLDL prevents the effects of enzalutamide on AR and AR-V7 expression in CRPC cell lines. Western blot analysis of AR and AR-V7 on C4-2B and 22Rv1 cells co-treated with enzalutamide [20 μM]/oxLDL [50 μg/mL]. The data represent the means ± S.D. of three independent experiments analyzed by one-way analysis of variance and Dunnett’s post-test. *** *p* ≤ 0.001, ** *p* ≤ 0.01, * *p* ≤ 0.05, ns: non-significant statistical difference).

The activation of NF-κB and STAT3 signaling pathways and the overexpression of AR, AR-V7, PSA, and IL-6 have been recognized as markers and inducers or effectors (AR- AR-V7) of enzalutamide resistance in CRPC [49,57]. Our results indicate that LOX-1 activation by oxLDLs induces the axis ROS/NF-KB/IL-6/STAT3 with the consequent overexpression of AR and AR-v7. We hypothesized that the overexpression of AR and AR-V7 could decrease the cytotoxic effect of enzalutamide, an antagonist drug of AR. Our results showed that oxLDL significantly increases the surviving fraction and the IC_50_ for enzalutamide by 1.8- and 2.7-fold in C4-2B and 22Rv1 cells, respectively (Figure 6). Moreover, LOX-1 knockdown in C4-2B and 22Rv1 cells prevented the effects of oxLDL, even displaying increased sensitivity to enzalutamide and higher cytotoxic effect of co-treatment with oxLDL/enzalutamide (Figure 6A,B). In this regard, we observed that enzalutamide treatment for 24 h decreased AR and AR-V7 expression in C4-2B and 22RV-1; however, this effect was prevented by oxLDLs/enzalutamide co-treatments (Figure 6A,B), suggesting that the cytotoxicity-lowering effects of enzalutamide mediated by oxLDLs are mediated by the increase in the expression of classical markers of enzalutamide resistance, such as AR and AR-V7 in C4-2B and 22RV-1 cells. 

## 3. Discussion

LOX-1 receptor and oxLDLs are associated with the development and progression of several pathologies, such as atherosclerosis, obesity, type 2 diabetes, and different cancers [22,23,24,25,26,27,28,29,30,31,32]. Notably, in CaP, we have previously shown that LOX-1 activation by oxLDLs stimulates tumor angiogenesis [31], epithelial-mesenchymal transition, and tumorigenic potential, being determinant for tumor growth [32], which could explain why obese patients with CaP progress to more malignant stages in a shorter period of time compared to patients with average weight [32]. In atherosclerosis and endothelial dysfunction, activation of LOX-1 stimulates the NF-κB signaling pathway to promote the expression of proinflammatory cytokines, such as IL-1b, TNF-α, MCP1, IL-8, and IL-6 [58]. Among these cytokines, IL-6 is particularly relevant because it induces the activation of STAT3, a critical factor in the development of CRPC and enzalutamide resistance [49,50,59]. In the present work, we demonstrated that oxLDL/LOX-1 increases ROS levels and activates NF-κB signaling pathway, inducing the secretion of IL-6 and the activation of STAT3 in CRPC cell lines. These data indicate that atherosclerosis and CRPC, two very different diseases at first sight, share pathological mechanisms at the molecular level, at least regarding the oxLDL/LOX-1 axis. Furthermore, oxLDL/LOX-1 increases AR and AR-V7 protein levels, two important markers for enzalutamide resistance, which explains the increase in IC_50_ for enzalutamide in CRCP cell lines. The ROS generation induced by oxLDL/LOX-1 has been reported in many studies associated with atherosclerosis, endothelial dysfunction, and macrophage activation [60]. We extended these findings to human CRPC cell lines in the present study. In endothelial cells, high concentrations of oxLDL are cytotoxic and induce cell death [61]. In contrast, low concentrations of oxLDL induce oxidative stress through stimulation of NAPDH oxidase, increased ROS production, and activation of NF-κB, which promotes the secretion of pro-inflammatory cytokines [62]. In contrast, breast cancer or CaP cells do not show significant changes in cell viability at a high concentration of oxLDLs. Moreover, previous studies by our group showed that oxLDLs at concentrations up to 100 μg/mL increased cell proliferation [63,64]. In this sense, high concentrations of oxLDLs could play an important role in the activation of several processes associated with tumor progression. 

Several authors have described that the activation of NF-κB by ROS induces IκBα phosphorylation-dependent or independent of IKK [65,66,67]. NF-κB signaling pathway induces IL-6 expression, which plays an essential role in prostate cancer progression, the development of CRPC, and enzalutamide resistance [51,68]. The overexpression of IL-6 increases the resistance of prostate cancer cells mainly through the JAK/STAT3 axis [69]. Thus, it has been demonstrated that inhibitors of the IL-6/STAT3 axis, such as galiellalactone or Stattic [70] and antibodies against IL-6 [71], among others, are viable alternatives to decrease the AR activity in prostate tissue and CaP. The ectopic expression of IL-6 in IL-6 negative LNCaP cells has been shown to significantly increase clonogenic and proliferative capacity, accompanied by JAK/STAT3 activation [72]. In this regard, CRPC cells secrete IL-6, which through an autocrine mechanism, induces enzalutamide resistance via the constitutive activation of STAT3, whereas its inhibition prevents or reverts enzalutamide resistance [51]. Likewise, the activation of NF-κB by oxLDL/LOX-1-ROS could induce the secretion of IL-6 observed in our study, which in turn may act in an autocrine fashion to stimulate STAT3 signaling, as it has been observed in other types of cancer [73]. Furthermore, our results showed that oxLDL promotes the expression of AR and its variant AR-V7, and a ROS, NF-κB, and STAT3 inhibitor prevented these effects. Similarly, oxLDL/LOX-1 increased AR and AR-V7 expression, promoted AR nuclear translocation, and increased PSA levels in the CRPC cell models used in our study. In the context of CRPC, it has been described that NF-κB can mediate the expression of enzalutamide resistance-related proteins, such as AR and AR-V7 [74]. Although there is no evidence linking oxLDL/LOX-1 to AR or AR-V7 expression, however, several reports have demonstrated that NF-κB activation is involved in the expression of these effectors [49,74,75,76]. In a model of benign prostatic hyperplasia, Austin et al. identified that NF-κB is important in the progression of this pathology and can lead to prostate cancer development [75]. In this same study, NF-κB activation significantly increased AR-V7 mRNA levels. Moreover, in the androgen-dependent cell line LNCaP or the androgen-independent cell line 22Rv1, AR and AR-V7 overexpression increased NF-κB activation, suggesting that there may be crosstalk between these pathways. Liu et al. demonstrated that the inhibition of NF-κB with melatonin prevents AR and AR-V7 overexpression in CaP cells [49]. Furthermore, in castration models, in which dihydrotestosterone concentration was significantly decreased, the high NF-κB activation is evidenced by phosphorylation of the p65 subunit [39]. Moreover, Nadiminty et al. demonstrated that in the androgen-sensitive cell line LNCaP, the over-activation of the non-canonical p52 NF-κB pathway also promoted AR-V7 expression [77]. In 22Rv1 cells, Kiliccioglu et al. observed that AR-V7 and AR expression was decreased upon inhibition of NF-κB with BAY 11-7082, reinforcing the idea that NF-κB is important in the overexpression of AR and AR-V7, two enzalutamide resistance markers [78]. 

Our results demonstrated that oxLDL treatments decrease enzalutamide cytotoxicity, an effect that is prevented when LOX-1 is silenced. These results confirm that the expression of enzalutamide resistance markers, such as AR and AR-V7, are associated with oxLDL-mediated LOX-1 activation. Our analysis demonstrated that CRPC cells co-treated with oxLDLs, and enzalutamide maintain the expression of AR and AR-V7 at the level of cells without enzalutamide treatment, strongly supporting that oxLDL decreases enzalutamide cytotoxicity mediated by LOX-1 activation.

Based on this evidence, it is reasonable to propose that oxLDL/LOX-1-mediated AR and AR-V7 expression in CRPC cells is a result of the NF-κB pathway activation. However, it is relevant to note that this effect could occur directly through oxLDL/LOX-1/ROS/NK-kB/AR axis activation or indirectly through the overexpression and secretion of proinflammatory cytokines, such as IL-6, which in turn act in an autocrine fashion to activate STAT3/AR axis. From this perspective, even if either of these events could justify the AR and AR-V7 overexpression, LOX-1 activation should induce an increase in the IC_50_ of AR-targeting drugs, such as enzalutamide. Thus, our results in conjunction with other investigations, demonstrate a relevant role of LOX-1 in cancer progression. These allow us to propose LOX-1 as a target in cancer therapy or, as in our study, as a target for the prevention of the resistance to enzalutamide in CRPC. In this regard, statins, an HMG-CoA reductase inhibitor, positively prevent cancer progression [79]. Likewise, it has been determined that they prevent LOX-1 expression and that statins, such as lovastatin would directly antagonize the binding of oxLDLs to LOX-1 [80]. Thus, FDA-approved drugs such as statins, could be recommended to prevent enzalutamide resistance in CRPC patients due to their antagonistic effect on LOX-1, a receptor that promotes antiandrogen resistance through the overexpression of AR and AR-v7.

In conclusion, treating CRPC patients is a complex process involving using different drugs for its control. However, resistance to drugs used in CRPC, such as enzalutamide, is observed in a large percentage of patients. Our results showed for the first time that LOX-1 and oxLDLs, two critical factors associated with pathologies of lipid metabolism and CaP tumor progression, are also determinants in the resistance to enzalutamide in human CRPC cell lines through NF-κB or STAT3 signaling pathway and AR and AR-V7 overexpression. 

## 4. Materials and Methods

### 4.1. Cell Culture

Human prostate cancer cells C4-2B and 22RV1 were grown in RPMI 1640 medium, supplemented with 2 mM L-glutamine (Hyclone), 10% fetal bovine serum, and 1% penicillin-streptomycin (GIBCO). The cell lines were maintained at 37 °C with 5% CO2. The C4-2B and 22RV1 cell lines are cell lines widely used in castration-resistant prostate cancer research. The C4-2B cell line is from LNCaP (androgen sensitive), co-inoculated in a nude mouse with human fibroblasts derived from osteosarcoma. The nude mice were castrated after eight weeks of incubation, and castration-resistant tumor cells (C4 cells) were isolated and characterized. The process was repeated to obtain the C4-2B cell line. The 22RV1 cell line is derived from xenograft that was serially propagated in mice after castration-induced regression and relapse of the parental, androgen-dependent CWR22 cell line xenograft. Both cell lines are androgen-independent but can respond to testosterone or DHT, and are able to secrete PSA. The cell lines express AR and its variant AR-V7. In this sense, it has been reported that 22RV1 expresses high levels of AR-V7 and other variants compared to C4-2B [81,82]. 

### 4.2. OxLDL Preparation

The LDL isolation and oxLDL generation were carried out, according to our previous reports [31,32]. The oxLDL was extensively dialyzed against PBS and stored at 4 °C. 

### 4.3. siRNA Transfection

siRNA against LOX-1 were acquired from ThermoFisher Scientific (S9842). For silencing the LOX-1 RNA expression, C4-2B and 22RV1 prostate cancer cell lines were transfected with 150 pmol of siRNA/LOX-1 or siRNA/control using Lipofectamine 3000 (Thermo Fisher Scientific, Waltham, MA, USA). Two days post-transfection, LOX-1 silencing was verified by Western blot. 

### 4.4. Immunodetection

Western blot and immunostaining were performed using standard protocols [26]. Anti-LOX-1 (LOX19-22) sc-66155, anti-AR (N-20) sc-816, anti-GAPDH (0411) sc-47724, anti-NF-kB p65 C-20) sc-372 and anti-IκB-alfa (C-21) sc-371, and anti-STAT3 (c-20) sc-482 antibodies were obtained from Santa Cruz Biotechnology. Anti-p-p65 s536 7F1, anti-p-IκB-alfa S32/S36 5A5, androgen receptor (AR-V7 Specific), and anti-p-STAT3 (Y705) (D3A7) antibodies were obtained from Cell Signaling. For Western blot, the anti-mouse IgG-Alexa Fluor 680 and anti-rabbit IgG-Alexa Fluor 790 secondary antibodies were obtained from Jackson ImmunoResearch. Western blots were analyzed using LICOR-CLX. An anti-rabbit IgG/FITC secondary antibody from Jackson ImmunoResearch was used for immunofluorescences. Photographs were obtained by confocal microscopy (Olympus IX81, Japan). To determine IL-6 concentration from supernatants, the human ELISA Kit (D6050, R&D) was used according to manufacturer’s instructions.

### 4.5. Real Time PCR

Real-time PCR for LOX-1 (Forward 5′- AGATCCAGACTGTGAAGGACCAGC-3′, reverse 5′- CAGGCACCACCATGGAGAGTAAAG -3′) and prostate-specific antigen (PSA) (Forward 5′- GCATTACCGGAAGTGGATCAAGGA-3′, reverse 5′- TTGAGTCTTGGCCTGGTCATTTCC-3′) were performed using KAPA one step SYBR^®^ FAST qPCR KIT (KAPA Biosystems, Wilmington, MA, USA) and Agilent AriaMx real-time PCR system equipment. The results were analyzed as 2^−ΔΔCT^ relative quantification. The comparative threshold cycle values were normalized for b-actin (Forward 5′-TGTACCCTGGCATTGCCGACAG-3′, Reverse 5′-ACGGAGTACTTGCGCTCAGGAG-3′.

### 4.6. ROS Determination

C4-2B and 22RV1 cells were transfected (20.000 cells) with siRNA control or siRNA LOX-1. Then, after 48 h, cells were seeded in 96-well black plates for fluorescence analysis. Cells were pre-incubated with 5 µM CM-H2DCFDA general oxidative stress indicator (C6827, Invitrogen) for 15 min at 37 °C and then treated with 50 µg/mL of oxLDLs during 3 h at 37 °C. As a control, C4-2B or 22RV1 cells were treated with 25 µM Trolox for 2 h and then incubated with 50 µg/mL oxLDLs for 3 h. 

### 4.7. Determination of NF-κB and STAT3 Activation 

Two experimental approaches were used to determine the effects of oxLDL/LOX-1 on NF-κB activation. On the one hand, C4-2B and 22RV1 cells transfected with siRNA control or siRNA LOX-1 for 48 h were treated with 50 µg/mL of oxLDLs for 1 h and the phosphorylation state of p65 and IκB-alpha subunit was analyzed by Western blot. On the other hand, transcriptional activity assays were performed using the luciferase reporter pHAGE NFKB-TA-LUC-UBC-dTomato-W (Catalog # 49335, Adggene) [61]. To this end, C4-2B and 22RV1 cells were co-transfected with a siRNA control or siRNA LOX-1 and the pHAGE NFKB-TA-LUC-UBC-dTomato-W plasmid. Luciferase activity was detected using the Synergy multi plate reader (BioTek) using the Luciferase assay system (E1500, Promega), normalizing against dTomato (excitation: 554 nm, emission: nm 581). For STAT3 activation, Tyr 705 phosphorylation was analyzed using Western blot. In this case, C4-2B and 22RV1 cells transfected with siRNA control or siRNA LOX-1 for 48 h were treated with 50 µg/mL of oxLDLs for 24 h. 

Furthermore, co-treatment with 50 µg/mL of oxLDLs and ROS inhibitor Trolox, three NF-κB inhibitors: triptolide (TLP) (Cayman Chemical # 11973), bay 11-7082 (BAY) (Cayman Chemical # 10010266), caffeic acid phenethyl ester (CAPE) (Cayman Chemical # 70750) and a chemical inhibitor of STAT3 activity Stattic (STAT3 inhibitor V, CAS 19983-44-9, Santa Cruz Biotechnology) were used in the ROS and pathways inhibition. 

### 4.8. Cytotoxic Assay Using Enzalutamide

Enzalutamide (CAS No. 915087-33-1, Cayman Chemical) was dissolved at 40 mM in DMSO and stored at −20 °C. The cytotoxic effects of enzalutamide on C4-2B and 22RV1 LOX-1 knockdown cells were determined by clonogenic assay. C4-2B and 22RV1 parental or LOX-1 knockdown cells were seeded on 6-well plates and pre-incubated with or without 50 µg/mL of oxLDL. Then, the cells were incubated with increasing concentrations of enzalutamide (0–50 mM) and cultured for 14 days at 37 °C. Colonies were stained with crystal violet, images were photo-documented using the LI-COR ODYSSEY CLX equipment, and total colony numbers were counted for each condition.

## Data Availability

Not applicable.
